# Vaccination with recombinant *Boophilus annulatus *Bm86 ortholog protein, Ba86, protects cattle against *B. annulatus *and *B. microplus *infestations

**DOI:** 10.1186/1472-6750-9-29

**Published:** 2009-03-31

**Authors:** Mario Canales, Consuelo Almazán, Victoria Naranjo, Frans Jongejan, José de la Fuente

**Affiliations:** 1 Instituto de Investigación en Recursos Cinegéticos IREC (CSIC-UCLM-JCCM), Ronda de Toledo s/n, 13071 Ciudad Real, Spain; 2 Facultad de Medicina Veterinaria y Zootecnia, Universidad Autónoma de Tamaulipas, Km. 5 carretera Victoria-Mante, CP 87000 Ciudad Victoria, Tamaulipas, Mexico; 3 Department of Veterinary Pathobiology, Center for Veterinary Health Sciences, Oklahoma State University, Stillwater, OK 74078, USA; 4 Utrecht Centre for Tick-borne Diseases (UCTD), Department of Infectious Diseases and Immunology, Faculty of Veterinary Medicine, Utrecht University, Yalelaan 1, 3584CL, Utrecht, The Netherlands; 5 Department of Veterinary Tropical Diseases, Faculty of Veterinary Science, University of Pretoria, Private Bag X04, 0110, Onderstepoort, South Africa

## Abstract

**Background:**

The cattle ticks, *Boophilus *spp., affect cattle production in tropical and subtropical regions of the world. Tick vaccines constitute a cost-effective and environmentally friendly alternative to tick control. The recombinant *B. microplus *Bm86 protective antigen has been shown to protect cattle against tick infestations. Recently, the gene coding for *B. annulatus *Bm86 ortholog, Ba86, was cloned and the recombinant protein was secreted and purified from the yeast *Pichia pastoris*.

**Results:**

Recombinant Ba86 (Israel strain) was used to immunize cattle to test its efficacy for the control of *B. annulatus *(Mercedes, Texas, USA strain) and *B. microplus *(Susceptible, Mexico strain) infestations. Bm86 (Gavac and Mozambique strain) and adjuvant/saline were used as positive and negative controls, respectively. Vaccination with Ba86 reduced tick infestations (71% and 40%), weight (8% and 15%), oviposition (22% and 5%) and egg fertility (25% and 50%) for *B. annulatus *and *B. microplus*, respectively. The efficacy of both Ba86 and Bm86 was higher for *B. annulatus *than for *B. microplus*. The efficacy of Ba86 was higher for *B. annulatus *(83.0%) than for *B. microplus *(71.5%). The efficacy of Bm86 (Gavac; 85.2%) but not Bm86 (Mozambique strain; 70.4%) was higher than that of Ba86 (71.5%) on *B. microplus*. However, the efficacy of Bm86 (both Gavac and Mozambique strain; 99.6%) was higher than that of Ba86 (83.0%) on *B. annulatus*.

**Conclusion:**

These experiments showed the efficacy of recombinant Ba86 for the control of *B. annulatus *and *B. microplus *infestations in cattle and suggested that physiological differences between *B. microplus *and *B. annulatus *and those encoded in the sequence of Bm86 orthologs may be responsible for the differences in susceptibility of these tick species to Bm86 vaccines.

## Background

*Boophilus *spp. (recently considered a synonym of *Rhipicephalus (Boophilus) *spp.) ticks are distributed in tropical and subtropical regions of the world with range expansion for some species due to changes in climatic conditions [1-3]. Infestations with the cattle tick, *Boophilus microplus*, economically impact cattle production by reducing weight gain and milk production, and by transmitting pathogens that cause babesiosis (*Babesia bovis *and *B. bigemina*) and anaplasmosis (*Anaplasma marginale*) [4]. *B. annulatus *is present in regions of Asia, Latin America and Africa [2] where it may also affect cattle production and vector pathogens.

Acaricide application constitutes a major component of integrated tick control strategies [5]. However, use of acaricides has had limited efficacy in reducing tick infestations and is often accompanied by serious drawbacks, including the selection of acaricide-resistant ticks, environmental contamination and contamination of milk and meat products with drug residues [5]. All of these issues reinforce the need for alternative approaches to control tick infestations such as the use of hosts with natural resistance to ticks, pheromone-impregnated decoys for attracting and killing ticks, biological control agents and vaccines [6-8].

In the early 1990s, vaccines were developed that induced immunological protection of vertebrate hosts against tick infestations. These vaccines contained the recombinant *B. microplus *Bm86 gut antigen [8-12]. Two vaccines using recombinant Bm86 were subsequently registered in Latin American countries (Gavac) and Australia (TickGARD) during 1993–1997 [13]. These vaccines reduce the number of engorging female ticks, their weight and reproductive capacity. Thus the greatest vaccine effect was the reduction of larval infestations in subsequent generations. Vaccine controlled field trials in combination with acaricide treatments demonstrated that an integrated approach resulted in control of tick infestations while reducing the use of acaricides [12-14]. These trials demonstrated that control of ticks by vaccination has the advantages of being cost-effective, reducing environmental contamination and preventing the selection of drug resistant ticks that result from repeated acaricide application. In addition, these vaccines may also prevent or reduce transmission of pathogens by reducing tick populations and/or affecting tick vectorial capacity [13-15].

Controlled immunization trials have shown that *B. microplus *Bm86-containing vaccines also protect against related tick species, *B. annulatus *and *B. decoloratus *[16-18]. However, *B. microplus *strain-to-strain variations in the susceptibility to Bm86 vaccination have been reported and the efficacy of the Bm86 vaccine is higher against *B. annulatus *than against *B. microplus *strains [6,16-18]. These results suggested that Bm86 sequence and/or tick physiological differences may influence the efficacy of the vaccine in *Boophilus *spp. [18-22].

Recently, the gene coding for *B. annulatus *Bm86 ortholog, Ba86, was cloned from an Israeli tick strain and the recombinant protein was secreted and purified from *Pichia pastoris *[23]. The Bm86 and Ba86 proteins showed over 90% similarity and immune cross-reactivity [23]. However, only cattle vaccination and tick infestation experiments could evaluate the efficacy of Ba86 against *B. annulatus *and *B. microplus *infestations and address the question of whether differences in the susceptibility to Bm86 vaccines between these tick species are due to sequence polymorphisms, physiological characteristics of the ticks or both.

In the experiments reported herein cattle were vaccinated with the recombinant Bm86 and Ba86 antigens and infested with *B. annulatus *and *B. microplus *to (i) evaluate the efficacy of recombinant Ba86 for the control of *B. annulatus *and *B. microplus *infestations and (ii) to provide evidence of whether sequence polymorphisms, tick physiological differences or both may account for differences in the efficacy of Bm86 vaccines against *Boophilus *spp. infestations.

## Results and Discussion

### The vaccination with recombinant Ba86 and Bm86 protected cattle against *B. annulatus *and *B. microplus *infestations

**Figure 1:**
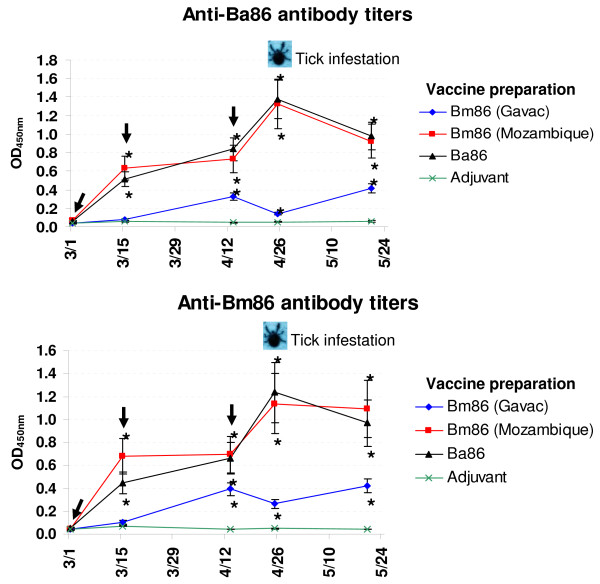
**Antibody response in vaccinated cattle**. Bovine serum antibody titers to recombinant Ba86 (Israeli strain) and Bm86 (Mozambique strain) antigens were determined by ELISA in cattle vaccinated with Bm86 (Gavac, Cuban Camcord strain), Bm86 (Mozambique strain), Ba86 (Israeli strain) and adjuvant/saline control. Antibody titers in immunized cattle were expressed as the OD_450 nm _value for the highest serum dilution (1:1000) and compared between vaccinated and control cattle using an ANOVA test (*P < 0.05). The time of vaccination shots (arrows) and tick infestation are indicated.

**Figure 2:**
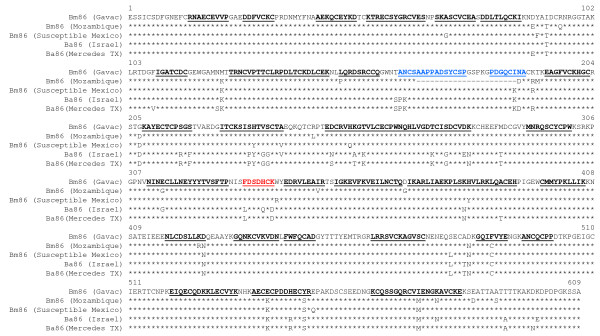
**Sequence comparison of recombinant Bm86 and Bm86 antigens**. The protein sequences of the Bm86 (Cuban Camcord in Gavac), Bm86 (Mozambique), Bm86 (Susceptible, Mexico), Ba86 (Israeli strain) and Ba86 (Mission, TX) strains were aligned and the antigenic peptides (≥ 7 residues) predicted using the method of Kolaskar and Tongaonkar [29], with a reported accuracy of about 75% . The predicted antigenic peptides are underlined. The antigenic peptide present in Bm86 but absent in Ba86 (Israel strain) is shown in underlined red letters. The antigenic peptides absent in the Bm86 (Mozambique strain) sequence are shown in underlined blue letters.

This is the first report on the protective capacity of recombinant Ba86 for the control of cattle tick infestations. To evaluate the protective capacity of Ba86 against *B. microplus *and *B. annulatus *infestations, cattle were vaccinated with the recombinant protein and compared to cattle vaccinated with two Bm86 preparations and adjuvant/saline control. The vaccinated animals but not the controls developed antibodies against recombinant proteins (Fig. 1). The antibody titers were similar for all groups when measured against Ba86 and Bm86 antigens, thus reflecting the presence of common antigenic epitopes between both proteins (ref. [23] and Fig. 2). The antibody titers in cattle vaccinated with Ba86 and Bm86 (Mozambique strain) were similar and higher than those in animals vaccinated with Bm86 (Gavac) (Fig. 1). As in previous experiments [11,20], except for animals vaccinated with Bm86 (Gavac), antibody titers increased after successive vaccinations and reached a peak two weeks after the third vaccination shot. The differences in the antibody response elicited by Bm86 (Gavac) and Bm86 (Mozambique strain) antigens could be attributed to differences in vaccine preparations (the Bm86 in Gavac was expressed in *P. pastoris *as membrane-bound while the Bm86 of the Mozambique strain was secreted in *P. pastoris*; ref. 23] and/or to other unknown factors such as cattle physiological status that have been demonstrated to affect cattle antibody response to Bm86 [14]. Additionally, as discussed bellow, Bm86 polymorphisms between Gavac and Mozambique strain antigens may affect antigen processing and immune response after vaccination [19].

The vaccination with recombinant Ba86 protected cattle against *B. microplus *and *B. annulatus *infestations (Tables 1 and 2). As in previous experiments with recombinant Bm86 preparations, tick vaccines decreased the number and weight of adult female ticks, oviposition and egg fertility [9,11,12,14,16,18,20-24]. The efficacy of both Ba86 and Bm86 vaccines was higher for *B. annulatus *than for *B. microplus *(Tables 1 and 2). The efficacy of Ba86 was higher for *B. annulatus *(83.0%) than for *B. microplus *(71.5%) while the efficacy of Bm86 (Gavac; 85.2%) but not Bm86 (Mozambique strain; 70.4%) was higher than that of Ba86 (71.5%) on *B. microplus*. However, the efficacy of Bm86 vaccines was higher than that of Ba86 for *B. annulatus *and similar to previous reports showing close to 100% efficacy of Bm86 for the control of *B. annulatus *infestations [16,18]. The efficacy of Bm86 vaccine preparations against *B. microplus *was within the range reported in previous experiments with other tick strains [12,17-19,24].

**Table 1:** Control of *B. microplus *infestations in cattle vaccinated with the recombinant Ba86 and Bm86 preparations.

	***Boophilus microplus *(Susceptible; Mexico strain)**				
	
	**Percent reduction (vaccinated/control)^b^**				**E^c^**
**Experimental group^a^**	DT	DW	DO	DF	
Ba86 (Israeli strain)	40% (506 ± 96)*	15% (252 ± 14)*	5% (104 ± 11)	50% (0.2 ± 0.04)*	71.5%

Bm86 (Gavac; Cuban Camcord strain)	59% (348 ± 99)*	15% (253 ± 9)*	28% (78 ± 8)*	50% (0.2 ± 0.03)*	85.2%

Bm86 (Mozambique strain)	22% (655 ± 172*)	17% (245 ± 14)*	24% (83 ± 11)*	50% (0.2 ± 0.04)*	70.4%

Adjuvant/saline control	841 ± 94	297 ± 19	109 ± 10	0.4 ± 0.0	---

^a^Cattle were randomly assigned to experimental groups (N = 5), vaccinated and challenged with *B. microplus *and *B. annulatus *larvae.

^b^The percent reduction was calculated with respect to the control group: DT, % reduction in tick infestation; DW, % reduction in tick weight; DO, % reduction in oviposition; DF, % reduction in egg fertility. In parenthesis are shown the average ± S.E. for adult female tick number, tick weight, oviposition and egg fertility and were compared by Student's t-test with unequal variance between vaccinated and control groups. (*P < 0.05).

^c^Vaccine efficacy (E) was calculated as 100 [l-(CRT × CR0 × CRF)], where CRT, CRO and CRF are the reduction in the number of adult female ticks, oviposition and egg fertility as compared to the control group, respectively.

**Table 2:** Control of *B. annulatus *infestations in cattle vaccinated with the recombinant Ba86 and Bm86 preparations.

	***Boophilus annulatus *(Mission, TX strain)**				
	
	**Percent reduction (vaccinated/control)^b^**				**E^c^**
**Experimental group^a^**	DT	DW	DO	DF	
Ba86 (Israeli strain)	71% (217 ± 128)*	8% (285 ± 17)	22% (89 ± 10)*	25% (0.3 ± 0.1)*	83.0%

Bm86 (Gavac; Cuban Camcord strain)	95% (34 ± 33)*	54% (141 ± 59)*	66% (39 ± 17)*	75% (0.1 ± 0.1)*	99.6%

Bm86 (Mozambique strain)	99% (4 ± 2)*	23% (240 ± 62)	25% (85 ± 24)*	50% (0.2 ± 0.1)*	99.6%

Adjuvant/saline control	750 ± 127	310 ± 52	114 ± 8	0.4 ± 0.04	---

^a^Cattle were randomly assigned to experimental groups (N = 5), vaccinated and challenged with *B. microplus *and *B. annulatus *larvae.

^b^The percent reduction was calculated with respect to the control group: DT, % reduction in tick infestation; DW, % reduction in tick weight; DO, % reduction in oviposition; DF, % reduction in egg fertility. In parenthesis are shown the average ± S.E. for adult female tick number, tick weight, oviposition and egg fertility and were compared by Student's t-test with unequal variance between vaccinated and control groups. (*P < 0.05).

^c^Vaccine efficacy (E) was calculated as 100 [l-(CRT × CR0 × CRF)], where CRT, CRO and CRF are the reduction in the number of adult female ticks, oviposition and egg fertility as compared to the control group, respectively.

### Polymorphisms in Bm86 orhtologs and physiological differences between *B. annulatus *and *B. microplus *may account for differences in the efficacy of Bm86 vaccines

The fact that both Ba86 and Bm86 vaccines had a higher efficacy for *B. annulatus *than for *B. microplus *suggested a tick species-specific effect that resulted in higher susceptibility of *B. annulatus *to vaccination. This effect may be related to tick physiological processes such as feeding and digestion. For example, a higher amount of blood ingestion or a lesser protease activity in the gut of *B. annulatus *would result in an increase in the number of antibody-antigen interactions and vaccine efficacy. A direct correlation between antibody titers and vaccine efficacy has been demonstrated for Bm86-based vaccines [14,24]. Further experiments would have to be conducted to address this important issue by comparing the amount of ingested blood using artificial feeding systems and protease and antibody gut concentration in feeding ticks between different *Boophilus *species and strains.

However, two results suggested that polymorphisms in Bm86 orthologs may also contribute to differences in vaccine efficacy between *B. annulatus *and *B. microplus*: (i) Despite lower antibody titers in vaccinated cattle, vaccine efficacy on *B. microplus *was higher for Bm86 (Gavac; Cuban Camcord strain) than for Bm86 (Mozambique strain) and (ii) the efficacy of Bm86 vaccines was higher than that of Ba86 on *B. annulatus*.

The analysis of Bm86 protein sequences showed that the antigen in the Mexican (Susceptible) strain used for infestation was 97% homologous to the sequence in Gavac Cuban Camcord strain but 93% homologous to the sequence of the Mozambique strain (Fig. 2). These differences in the sequence of Bm86 may affect the efficacy of Bm86 vaccines in different strains [19]. For example, two of the predicted antigenic peptides in Bm86 (Gavac) were located on a deletion in the Bm86 (Mozambique strain) sequence but conserved in the Bm86 (Susceptible, Mexico) strain sequence (Fig. 2). The efficacy of the Ba86 vaccine on *B. microplus*, which was slightly higher (71.5%; Table 1) than that of the Bm86 (Mozambique strain) vaccine (70.4%; Table 1) also supports this hypothesis because the homology to the Bm86 sequence in the Mexican strain used for infestation was also higher for Ba86 (94%) than for Bm86 (Mozambique strain; 93%). However, as discussed above, differences in the production of Gavac and the Mozambique strain vaccines [23] together with cattle physiological factors [14] and antibody isotype composition [25] may also account for differences in vaccine efficacy.

Despite the possibility of higher susceptibility of *B. annulatus *to vaccination discussed above, the analysis of predicted antigenic regions in Ba86 and Bm86 also suggested an effect of protein sequence on vaccine efficacy. Several predicted antigenic regions were polymorphic between Bm86 and Ba86 (Fig. 2). Furthermore, Bm86 contained one predicted antigenic region not present in Ba86 (Israel strain) but present in Ba86 (Mercedes, Texas strain) (Fig. 2). The predicted antigenic regions may contain protective epitopes and thus could be involved in eliciting a protective response after vaccination. Therefore, polymorphisms in these regions could explain, at least for some Ba86 antigens, the higher efficacy of Bm86 vaccines for the control of *B. annulatus*.

## Conclusion

The results reported herein demonstrated the efficacy of recombinant Ba86 for the control of *B. annulatus *and *B. microplus *infestations in cattle. These experiments also expanded the results of the efficacy of Bm86 vaccines by including protection against cattle infestation by new strains of *B. anulatus *(Mercedes, Texas, USA) and *B. microplus *(Susceptible, Mexico). Finally, these results suggested that physiological differences between *B. microplus *and *B. annulatus *and those encoded in the sequence of Bm86 orthologs may be responsible for the differences in susceptibility of these tick species to Bm86 vaccines.

## Methods

### Tick strains

The *B. microplus *(Susceptible, CENAPA, Mexico strain) and *B. annulatus *(Mercedes, Texas, USA strain) ticks were obtained from laboratory colonies maintained at the University of Tamaulipas, Mexico. Originally, these tick strains were collected from infested cattle in Tapalpa, Jalisco, Mexico and Mercedes County, Texas, USA for *B. microplus *and *B. annulatus*, respectively. Ticks were maintained during two years at the facilities of the Faculty of Veterinary Medicine, University of Tamaulipas, where several generations of tick larvae were fed on cows and collected until repletion to allow for oviposition and hatching in humidity chambers at 12 hr light: 12 hr dark photoperiod, 22–25°C and 95% relative humidity. Larvae were 15 days of age at the time of infestations.

### Vaccine formulations

The recombinant Ba86 (Israeli strain) and Bm86 (Mozambique strain) were secreted in *P. pastoris *and purified as reported previously [23]. Protein adjuvation was made by mixing a solution of anhydromannitoletheroctodecenoate (Montanide ISA 50 V; Seppic, Paris, France) with the recombinant protein solution in batch-by-batch processes using a high-speed mixer Heidolph DIAX 900 (Heidolph Elektro, Kelheim, Germany) at 8,000 rpm and the vaccine was filled manually under sterile conditions in glass bottles of 20 ml (Wheaton, Millville, NJ, USA) at a concentration of 100 μg/2 ml dose. Quality controls were made by testing mechanical and thermal stability of vaccine emulsions as described by Canales et al. [26]. The commercial Bm86 (Cuban Camcord strain) vaccine (Gavac, Revetmex, Mexico City, Mexico) also contains 100 μg/2 ml dose of *P. pastoris*-derived purified recombinant protein formulated as described above.

### Cattle immunization with recombinant proteins and tick infestations

Five crossbred calves per group were each immunized with 3 doses (weeks 1, 3 and 7) containing 100 μg/dose of purified recombinant proteins formulated as described above. Negative controls were injected with adjuvant/saline alone. Cattle were injected intramuscularly with 2 ml/dose using a 5 ml syringe and an 18G needle. Twelve days after the last immunization, cattle in vaccinated and control groups were infested with 10,000 *B. annulatus *(Mercedes, Texas, USA strain) and *B. microplus *(Susceptible, Mexico strain) larvae/animal applied individually to each animal in separate cotton cells attached to the back of the animals. Cattle were cared for in accordance with standards specified in the Guide for Care and Use of Laboratory Animals.

### Data collection and evaluation

Adult female ticks dropping from cattle were daily collected, counted and weighted. All the collected adult female ticks were assessed for oviposition and egg fertility [27]. The personnel collecting the ticks were 'blinded' as to which group animals belonged. The efficacy of vaccine formulations was evaluated employing the following formulae [27].

Effect on the number of adult female ticks (DT) = 100 [l-(NTV/NTC)], where NTV is the number of adult female ticks in the vaccinated group and NTC is the number of adult female ticks in the control group.

Effect on tick weight (DW) = 100 [1-(WTV/WTC)], where WTV is the average adult female tick weight in the vaccinated group and WTC is the average adult female tick weight in the control group.

Effect on oviposition (DO) = 100 [1-(PATV/PATC)], where PATV is the average weight of the eggs per survived tick in the vaccinated group and PATC is the average weight of the eggs per survived tick in the control group.

Effect on egg fertility (DF) = 100 [1-(PPLOV/PPLOC)], where PPLOV is the average weight of the larvae per gram of eggs in the vaccinated group and PPLOC is the average weight of the larvae per gram of eggs in the control group.

Vaccine efficacy (E) was calculated as 100 [l-(CRT × CR0 × CRF)], where CRT = NTV/NTC, CR0 = PATV/PATC and CRF = PPLOV/PPLOC that represent the reduction in the number of adult female ticks, oviposition and egg fertility as compared to the control group, respectively.

A Student's t-test with unequal variance (P = 0.05) was used to compare the results of adult female tick number, tick weight, oviposition and egg fertility between vaccinated and control groups.

### Determination of serum antibody levels by ELISA

Before each immunization and 12 (before tick infestation) and 37 days after the last immunization, blood samples were collected from each calf into sterile tubes and maintained at 4°C until arrival at the laboratory. Serum was then separated after centrifugation and stored at -20°C. Serum antibody titers were determined using an antigen-specific indirect ELISA. Purified recombinant Bm86 (Mozambique strain) and Ba86 (Israeli strain) antigens (0.1 μg/well) were used to coat ELISA plates overnight at 4°C. Sera were serially diluted to 1:10, 1:100 and 1:1000 in PBST (PBS/0.5% Tween 20, pH 7.2) and 10% fetal bovine serum (Sigma). The plates were incubated with the diluted sera for 1 hr at 37°C and then incubated with 1:10,000 rabbit anti-bovine IgG-HRP conjugates (Sigma) for 1 hr at 37°C. The color reaction was developed with 3,3',5,5'-tetramethylbenzidine (Sigma) and the OD_450 nm _was determined. After incubation the plates were washed with PBST. Antibody titers were considered positive when they yielded an OD_450 nm _value at least twice as high as the preimmune serum. Antibody titers in immunized cattle were expressed as the OD_450 nm _value for the highest serum dilution (1:1000) and compared between vaccinated and control cattle using an ANOVA test (P < 0.05).

### Sequence analysis

The sequences of the Ba86 (Mercedes, Texas, USA; Genbank accession number FJ456927) and Bm86 (Susceptible, Mexico; FJ456928) strains were determined as described previously [23]. The protein sequences were aligned with Ba86 (Israeli strain; ABY58969), Bm86 (Mozambique strain; ABY58968) and Bm86 (Cuban Camcord strain in Gavac; [11]) using the program AlignX (Vector NTI Suite V 8.0, InforMax, Invitrogen, Carlsbad, CA, USA) with an engine based on the Clustal W algorithm [28]. Antigenic peptides (=7 residues) were predicted using the method of Kolaskar and Tongaonkar [29], with a reported accuracy of about 75% .

## Authors' contributions

MC carried out the expression, fermentation, protein purification and vaccine formulation. CA conducted the vaccine trial and determined serum antibody levels by ELISA. VN determined the sequence of Ba86 and Bm86. JF did sequence analyses. JF and FJ conceived the study, and participated in its design and coordination and drafted the manuscript. All authors read and approved the final manuscript.
